# Towards High-Performance Photo-Fenton Degradation of Organic Pollutants with Magnetite-Silver Composites: Synthesis, Catalytic Reactions and In Situ Insights

**DOI:** 10.3390/nano14100849

**Published:** 2024-05-13

**Authors:** Katia Nchimi Nono, Alexander Vahl, Huayna Terraschke

**Affiliations:** 1 Institute of Inorganic Chemistry, Kiel University, Max-Eyth-Str. 2, 24118 Kiel, Germany; 2 Department of Inorganic Chemistry, Faculty of Science, University of Yaoundé, Yaoundé P.O. Box 812, Cameroon; 3 Department for Material Science, Kiel University, Kaiserstr. 2, 24143 Kiel, Germany; 4 Kiel Nano, Surface and Interface Science (KiNSIS), Christian-Albrechts-Platz 4, 24118 Kiel, Germany

**Keywords:** iron oxide, silver, nanocomposites, rhodamine B, photo-Fenton, luminescence, redox potential, pH values

## Abstract

In this study, Fe_3_O_4_/Ag magnetite-silver (MSx) nanocomposites were investigated as catalysts for advanced oxidation processes by coupling the plasmonic effect of silver nanoparticles and the ferromagnetism of iron oxide species. A surfactant-free co-precipitation synthesis method yielded pure Fe_3_O_4_ magnetite and four types of MSx nanocomposites. Their characterisation included structural, compositional, morphological and optical analyses, revealing Fe_3_O_4_ magnetite and Ag silver phases with particle sizes ranging from 15 to 40 nm, increasing with the silver content. The heterostructures with silver reduced magnetite particle aggregation, as confirmed by dynamic light scattering. The UV–Vis spectra showed that the Fe:Ag ratio strongly influenced the absorbance, with a strong absorption band around 400 nm due to the silver phase. The oxidation kinetics of organic pollutants, monitored by in situ luminescence measurements using rhodamine B as a model system, demonstrated the higher performance of the developed catalysts with increasing Ag content. The specific surface area measurements highlighted the importance of active sites in the synergistic catalytic activity of Fe_3_O_4_/Ag nanocomposites in the photo-Fenton reaction. Finally, the straightforward fabrication of diverse Fe_3_O_4_/Ag heterostructures combining magnetism and plasmonic effects opens up promising possibilities for heterogeneous catalysis and environmental remediation.

## 1. Introduction

A well-established strategy to mitigate the water supply problem in urban or remote areas is chemical water treatment. In fact, clean water can be obtained from wastewater by, for example, removing organic pollutants using advanced oxidation processes (AOPs) [1]. In order to reduce the consumption of energy and chemical reagents during wastewater treatment, the use of catalysts is being increasingly explored, with a particular interest in nanomaterials [2,3]. Among the various nanomaterials engineered for large-scale and industrial catalytic transformations, iron oxide nanoparticles have been extensively synthesised and studied [4]. Magnetite nanoparticles, with an inverse spinel structure of Fe^2+^ and Fe^3+^ ions with the chemical formula Fe_3_O_4_, are of particular interest as they exhibit high magnetic susceptibility combined with broad availability and low toxicity to both the environment and the human body. To date, a wide range of applications of Fe_3_O_4_ nanoparticles have been reported, including medicine, data storage and catalysis [5,6,7,8,9]. In the field of heterogeneous catalysis, Fe_3_O_4_ nanoparticles are particularly promising due to their magnetic properties. They can be easily removed from different media or mixtures by magnetic manipulation, allowing them to be reused in subsequent catalytic cycles. This recyclability is of paramount importance in heterogeneous catalysis experiments, where the attractiveness of a catalyst depends not only on its performance in chemical transformations but also on its ease of removal, cyclability and recyclability [5].

Specifically, Fe_3_O_4_ nanoparticles have found widespread application as catalysts for the degradation of organic pollutants through AOPs—in particular, the Fenton reaction [6]. In this reaction, H_2_O_2_ and Fe^2+^ ions form the highly reactive hydroxyl radical •OH. The Fenton reaction is one of the most potent AOPs due to the generation of reactive oxygen species (ROS) [6]. Fe_3_O_4_ nanoparticles with surface octahedral Fe^2+^ are active in Fenton-like catalytic oxidation and act as direct bandgap semiconductors activated by photon irradiation to promote the oxidation of organic dyes [7].

However, despite its advantages, the application of Fe_3_O_4_ in catalysis imposes a series of challenges. The catalytic activity of Fe_3_O_4_ nanoparticles results from Fe^2+^ ions increasing the oxidation potential of H_2_O_2_ [6], and this transformation is optimal in an acidic medium (pH = 3) [8]. This need for pH control is one of the limitations of the use of Fe_3_O_4_ for AOPs, as it not only adds to the operating costs but also contaminates the effluent and reduces the catalyst’s efficiency due to the partial dissolution of Fe^2+^ ions [9]. Moreover, Fe_3_O_4_ nanoparticles exhibit instability in water in the presence of dissolved oxygen species, impeding their facile removal from effluents and their industrial potential [10]. Several strategies have been proposed to prevent both the leaching of iron species and the oxidation of magnetite, including the immobilisation of Fe_3_O_4_ or the formation of composite materials [11].

In this context, noble elements such as silver and gold stand out as promising candidates, as they provide relative chemical inertness under adverse conditions. In fact, the association of noble metals with magnetite can improve the performance of the material in terms of photocatalysis, as well as providing other synergistic functionalities that go beyond the functionalities of the base materials. For example, with narrow bandgaps down to 2 eV, Fe_3_O_4_ nanoparticles have strong absorption in the visible range [6]. However, a small bandgap is also associated with fast electron–hole recombination. The formation of composite nanoparticles with silver can allow the trapping of electrons, thereby increasing the lifetime and reactivity of electron–hole pairs [12], while still exhibiting enhanced light absorption due to the local surface plasmon resonance (LSPR) that it exhibits [13,14]. In addition, the presence of oxygen vacancies in the semiconductor component, such as magnetite, further contributes to the photocatalytic activity of the composite nanoparticles. Oxygen vacancies serve as active sites for redox reactions and facilitate charge transfer processes, thereby promoting the generation and utilisation of ROS in photocatalytic reactions [15,16]. These effects, combined with the electron trapping capabilities of plasmonic nanoparticles, highlight the multiple mechanisms underlying the enhanced photocatalytic performance of composite materials [17,18,19].

The development of efficient nanocomposites for AOPs requires a thorough understanding of the transformations involved. Insightful information about parameters such as the pH and redox potential provides valuable perspectives on the reaction mechanisms, enabling the optimisation of nanomaterial-assisted oxidation processes. In situ studies under real conditions are powerful tools to explore and understand changes at each stage of a chemical transformation. While most previous in situ studies have focused on structural and electronic changes in solids, particularly at the reaction surfaces, these analyses typically require powerful equipment, such as synchrotron light sources. In contrast, the in situ monitoring of the pH, redox potential and photoluminescence (PL) in solutions can be easily performed in conventional laboratories. For example, in situ studies have been used extensively to monitor crystallisation processes [20,21,22] and in situ PL has been used to study the thermocatalytic conversion of 2-propanol to acetone using Co_3_O_4_ nanowires [23] and to monitor the photodegradation of organic dyes [24].

In this work, we explore the photocatalytic activity of Fe_3_O_4_/Ag nanocomposites by exploiting the magnetic properties and oxygen vacancies originating from the Fe_3_O_4_ phase and the plasmonic effect originating from the Ag phase. We describe the synthesis of composite catalysts through a very simple, mild, surfactant-free and straightforward synthetic procedure involving the reduction of silver ions using ascorbic acid in the presence of Fe_3_O_4_ nanoparticles prepared by co-precipitation. The combination of Fe_3_O_4_ nanoparticles and silver nanoparticles results in UVA-light-active catalysts for photo-Fenton-like processes. In addition, we present a novel approach by performing in situ monitoring of the photo-Fenton reactions using key parameters such as PL, the pH and the redox potential for the first time. The analysis of these data provides crucial insights into the transformations occurring during both the synthesis of Fe_3_O_4_/Ag nanocomposites and the degradation of rhodamine B (RhB), which serves as a model system for organic pollutants. Finally, the presence of silver plasmonic nanostructures accelerates H_2_O_2_ decomposition under UV LED irradiation.

## 2. Materials and Methods

### 2.1. Chemicals and Reagents

Analytical-grade FeCl_2_∙4H_2_O, H_2_O_2_ (30% (*w*/*w*) in H_2_O) and NaOH were purchased from Sigma Aldrich (Darmstadt, Germany). FeCl_3_∙6H_2_O, AgNO_3_, ascorbic acid and RhB were purchased from Merck (Darmstadt, Germany). Double-distilled water was used as the solvent.

### 2.2. Synthesis of Silver Magnetite Nanocomposites

In the first step, an aqueous solution of 15.8 mmol FeCl_3_∙6H_2_O with 10.14 mmol FeCl_2_∙4H_2_O in 50 mL distilled water was degassed with a nitrogen stream for 15 min. Then, 4 mL of NaOH solution (2 M) was added to the mixture over 5 min with vigorous stirring to reach pH = 10 until black particles were formed. The mixture was stirred at room temperature for 30 min. The solid particles formed were separated from the solvent using an external magnet and washed twice with distilled water until the pH was neutral. The synthesised material was again dispersed in 50 mL of water for the second step.

To combine the Fe_3_O_4_ nanoparticles with a plasmonic component, the freshly prepared Fe_3_O_4_ particles were dispersed in 50 mL of water using an ultrasonic bath. Different amounts of AgNO_3_ solution (0.4 M) were then added, ranging from 1 to 4 eq with respect to the estimated Fe^2+^ content (4 mL, 8 mL, 12 mL and 16 mL, respectively). The as-prepared compositions were labelled MS1, MS2, MS3 and MS4 for 1, 2, 3 and 4 eq silver nitrate added, respectively. The respective solutions were homogenised in an ultrasonic bath for 5 min. Finally, the stoichiometric amount of ascorbic acid solution (0.4 M) with respect to silver nitrate was added to the previous mixtures and the ultrasonic treatment continued for 15 min (4 mL, 8 mL, 12 mL and 16 mL, respectively). The resulting particles were washed three times with 50 mL of distilled water and separated from the solution using a magnet. This step is important to exclude non-magnetic species from the final product. The greyish particles were finally washed with ethanol (10 mL) before being dried in a vacuum oven at 50 °C for 12 h.

### 2.3. Characterisation of Nanoparticles

The synthesised Fe_3_O_4_ nanoparticles and composites were characterised by Fourier transform infrared (FTIR) spectroscopy, Raman spectroscopy, UV–Vis spectroscopy, powder X-ray diffraction and SEM/EDX analyses. The X-ray diffraction patterns were obtained using a Rigaku RINT 2000 diffractometer (Tokyo, Japan) with a MoK_α_ radiation source (at *λ* = 0.70930 Å). The UV–Vis spectra Varian Cary 5000 UV-Vis NIR (Melbourne, Australia) were obtained using the diffuse reflectance method with BaSO_4_ as a reference. The energy bandgap for direct and indirect transitions was calculated from the UV–Vis spectra using Tauc’s Equation (1).

(1) $$\omega^{2}\varepsilon ={\left(h\omega -E_{g}\right)}^{2}$$

with $\omega =\frac{2\pi }{\lambda }$.

Dynamic light scattering (DLS) measurements were performed using a Malvern Instruments Zetasizer Nano ZS (Worcestershire, UK) to determine the hydrodynamic diameter (DH) and zeta potential of the particles, together with the polydispersity index (PDI) of the suspension prepared by suspending 0.01 mg/mL of the particles in distilled water. Both D_h_ and the zeta potential were set to an automatic measurement duration with 3 runs and no delay between them at 25 °C. The specific surface area was determined by N_2_ sorption measurements using a BELSorp Max instrument (Kyoto, Japan). Samples were activated at 80 °C for 2 h under reduced pressure (<10 Pa).

Scanning electron microscopy (SEM) was performed using a scanning electron microscope Zeiss, Ultra Plus (Jena, Germany) with an in-lens detector and an acceleration voltage of 3 kV (at a working distance of 2.5 mm ± 0.1 mm). For the energy-dispersive X-ray (EDX) analysis, a Si drift detector Oxford Instruments, Ultim Max 65 (Jena, Germany) was used, and the SEM was operated at an acceleration voltage of 15 kV (at a working distance of 8.5 mm ± 0.1 mm).

### 2.4. RhB Degradation Experiments

The degradation of RhB by hydrogen peroxide H_2_O_2_ and the synthesised catalysts was assessed by UV–Vis absorption, in situ luminescence spectroscopy and pH and redox potential measurements. To ensure reproducibility, the experiments were performed in triplicate. Under dark conditions, i.e., in the absence of irradiation, RhB is not luminescent. Thus, the degradation was monitored by the variation in the absorbance of RhB recorded with a Varian Cary 5000 UV-Vis NIR spectrophotometer (Melbourne, Australia) in the presence of H_2_O_2_ (0.13 M) (4%) solution. The photodegradation of the RhB solution (10 mg L^−1^) and the photocatalytic activity of the nanoparticles were evaluated under 395 nm irradiation from a UV light-emitting diode (LED) from Sahllmann Photochemical Solutions (Bad Segeberg, Germany). The in situ luminescence was recorded with a portable spectrometer EPP2000 (StellarNet Inc., Tampa, FL, USA) equipped with a CCD detector attached to a fiber optic cable suspended above the reactor solution. To aid the dispersion of the particles, the suspension was stirred at 200 rpm throughout all catalytic reactions. The variations in the pH and redox potential values during photocatalysis were monitored using a Metrohm AG Titrando (Filderstadt, Germany).

## 3. Results and Discussion

### 3.1. Structure, Size and Morphology

**Figure 1:**
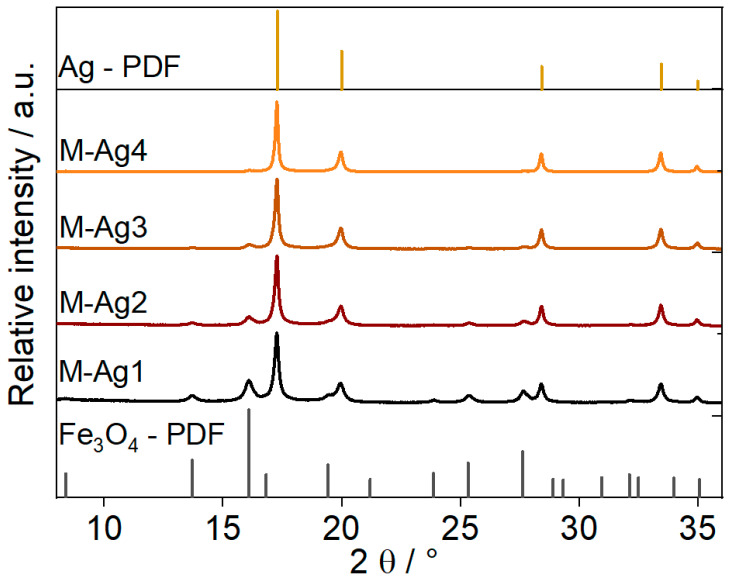
X-ray diffraction patterns of synthesised nanoparticles and nanocomposites compared with simulated diffraction patterns of Fe_3_O_4_ [25] and Ag [26].

The XRD patterns of the Fe_3_O_4_ nanoparticles and MSx composites are shown in Figure 1. The patterns are dominated by broad Bragg peaks, indicating the formation of small-sized particles. The Bragg peaks are compared to ICSD numbers 98-003-0860 [25] and 98-018-0878 [26] as references for Fe_3_O_4_ and Ag, respectively. The MS1 pattern shows the different diffraction peaks for Fe_3_O_4_ ((022), (113), (004), (224), (115), (044)) and for Ag ((111), (002), (022), (113) and (222)). Increasing the amount of silver nitrate reduces the significance of the Bragg peaks of Fe_3_O_4_. The XRD pattern for MS4 displays fewer diffraction peaks and seems to be mainly composed of silver. The crystallite size (D_XRD_) and lattice strain (*ε*) of the synthesised nanoparticles were calculated using the Scherrer equation D = 0.9*λ*/β cosθ and the equation *ε* = β/(4tanθ), respectively [27]. Using these equations, it was possible to estimate the crystallite size from the five most intense Bragg peaks of the materials (detailed in the Supplementary Materials).

The estimated crystallite sizes (Table 1) increase slightly from about 24 nm for MS1 to 39 nm for MS4, in good agreement with the higher silver equivalent in the solution. Conversely, a decrease in lattice strain is observed, which could be attributed to a decrease in defects and vacancies in the local structure with the formation of larger silver domains [28]. No reflexes other than those belonging to the cubic Fe_3_O_4_ and Ag phase are observed in the XRD patterns, indicating the formation and growth of pure Fe_3_O_4_ and Ag phases. The nominal ratios between Fe_3_O_4_ and Ag are in accordance with those estimated by integrating the reflex intensity (see Figure S5a–e) for MS1 and MS2, which yielded molar ratios of 1:1 and 1:2, respectively.

The particle size is a key parameter that has a significant impact on the properties of materials, especially for nanomaterials. The high surface-to-volume ratio of NPs is inversely proportional to the diameter of the particles [29]. Therefore, the smaller the particles, the greater their surface area and the more the particles can interact with substrates for targeted applications, including photocatalysis. However, for small Fe_3_O_4_ nanoparticles, the van der Waals attractive forces induce the formation of large aggregates, especially in the absence of surfactants or dipolar interactions [29]. Typically, although the crystallite size for Fe_3_O_4_ nanoparticles calculated from XRD data is very small, the DLS measurements show a very large hydrodynamic diameter of D_H_ = 409 nm for pure Fe_3_O_4_ nanoparticles, indicating the presence of multiple crystallites in one NP or, more likely, the formation of larger aggregates (see Figure S1). The addition of silver to the magnetic particles leads to a significant reduction in the hydrodynamic size from 409 to 91 nm for MS1. This substantial modification is mostly the result of the amplified electrostatic stabilisation in the composites, which offsets the van der Waals attraction that is predominant in iron oxide nanoparticles. Non-coated silver nanoparticles generally have a negative surface charge, as observed in previous studies [8]. Moreover, their formation on the surfaces of positively charged Fe_3_O_4_ nanoparticles can potentially stabilise the nanocomposites’ dispersion through repulsive electrostatic interactions [8]. It appears that the findings support the formation of heterostructures for MS1, with both Fe_3_O_4_ and Ag phases present. From MS1 to MS4, the stronger interparticle attraction, resulting in larger aggregates, may stem from the predominance of larger silver crystallites and their self-aggregation in the absence of surface functionalisation. The proposed variations and additional reversal in surface charge polarity are substantiated by the zeta potential measurements of iron oxide NPs and MSx NCPs outlined in Table 2. These outcomes demonstrate that pristine Fe_3_O_4_ NPs possess a positive surface charge (positive zeta potential values), whereas all composites exhibit negative surface charges (negative zeta potential values). It is important to note that the zeta potential measurements were performed in this work by dispersing the sample in distilled water, without adjusting the pH value, which may limit the quantitative comparability of the absolute values [30]. However, the consistent transition from positive zeta potentials in pristine Fe_3_O_4_ to negative in the composites remains a crucial observation and is indicative of underlying surface chemistry changes [31]. An increase in the average particle size at higher nominal concentrations of silver nitrate during synthesis was observed. This was due to the fact that the hydrodynamic size increased from MS1 to MS4 with the increase in the silver phase ratio.

**Figure 2:**
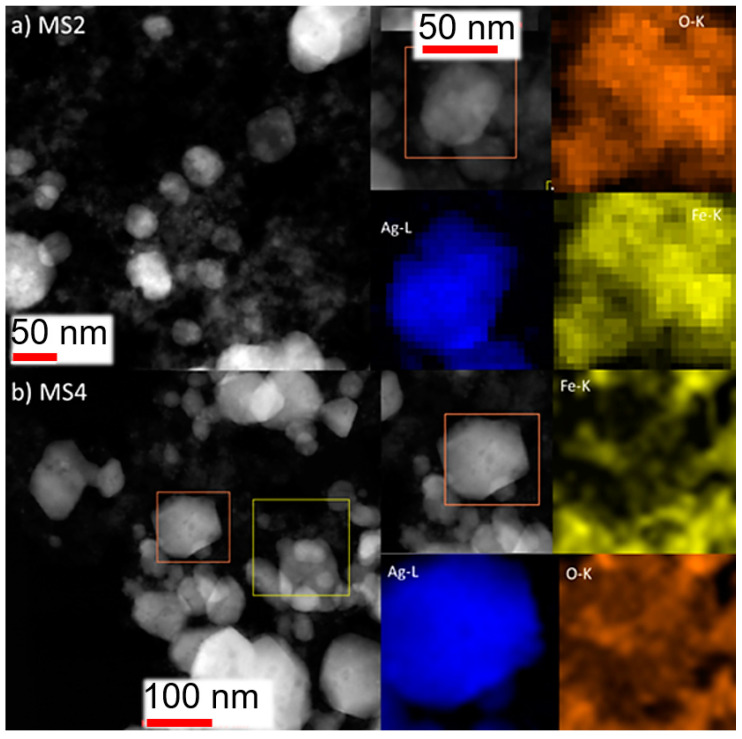
TEM images of MS2 and MS4.

The microstructural characteristics of the MSx nanocomposites were analysed by scanning electron microscopy (SEM), as shown in Figure S1. MS1 appeared predominantly as very small spherical particles that were highly aggregated. As the silver content increased, there was a marked increase in the diameters of the particles and their aggregation state. In MS2, MS3 and MS4, the particles were more dispersed and the distribution became increasingly bimodal, leading to the formation of interesting rods in MS4. Additional transmission electron microscopy (TEM) analysis of MS2 and MS4 provided further insights into the formation and growth of the composites with different silver ratios. The analysis revealed that small Fe_3_O_4_ particles formed island structures upon aggregation around non-spherical and some large Ag particles of approximately 50 nm in diameter (Figure 2).

The composition of the samples was further evaluated by energy-dispersive X-ray (EDX) analysis, as shown in Figure S12. Both iron and silver were detected, with the ratio increasing from MS1 to MS2 and then to MS4, with values of 0.5, 1.2 and 3.1, respectively.

Despite having similar morphologies, the specific surface areas of the four composites differ significantly. In particular, MS1 and MS2 have the highest values of the specific surface area, with S_bet_ values of 126.3 m^2^·g^−1^ and 79.7 m^2^·g^−1^, respectively. In contrast, MS3 and MS4 exhibit significantly smaller specific surface areas, with S_bet_ values of 39.3 m^2^·g^−1^ and 19.0 m^2^·g^−1^, respectively (refer to Table S6 in Supplementary Materials). The large active surface area and good porosity of MS1 and MS2 suggest that they may have superior catalytic performance due to the increased contact area between the catalyst and the reactants.

### 3.2. Spectroscopic Characterisation

The FTIR spectrum of the Fe_3_O_4_ NPs (see Figure S3 in the Supplementary Materials) shows absorption peaks at 3380 cm^−1^ and at 1300 and 1500 cm^−1^, which can be attributed to the stretching and bending of the O-H bond, typical of hydroxyl groups of water molecules on the surfaces of particles. The peak at 542 cm^−1^ corresponds to the Fe-O stretching mode of the tetrahedral site [32]. As the composite is formed, the absorption peak at 3380 cm^−1^ decreases dramatically and shifts to 3730 cm^−1^. Such a decrease may indicate an increase in silver content.

The comparison of the Raman spectra recorded for the studied materials (see Figure S3 in the Supplementary Materials) shows a contrast between the single magnetite phase, which has no absorption peaks below 1000 cm^−1^, and the nanocomposites. The absorption peak at 1039 cm^−1^ is assigned to the Ag vibrational mode [33], while the peak at 902 cm^−1^ corresponds to the vibration of Ag-O [34], originating from water molecules at the surface of the silver component of the particles, especially since the XRD patterns showed the absence of an AgO phase. This statement is also supported by the absence of Ag-O stretching mode Raman absorption at 230 cm^−1^ [35,36]. The intensity of the peaks is found to increase from MS1 to MS4, in agreement with the higher silver ratios. The peaks at ~550 cm^−1^ and ~1400 cm^−1^ are usually expected for pure magnetite-like materials [37].

**Figure 3:**
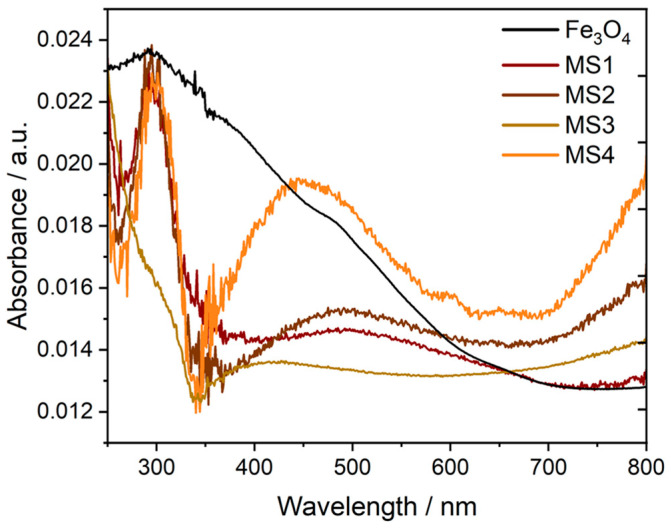
Diffuse reflectance spectra of Fe_3_O_4_ and composite nanoparticles. Reflectance measurements were converted into absorbance spectra using the Kubelka–Munk function [40].

The optical properties of the nanocomposites were analysed using UV–Vis absorption spectroscopy. This method, conducted through diffuse reflectance in the solid state, provided primarily qualitative insights. The reference Fe_3_O_4_ nanoparticles exhibited a broad absorption spectrum, primarily in the UV-B region. The composite spectra display a combination of the absorption characteristics of the Fe_3_O_4_ phase and the Ag phase, predominantly centred around 400 nm [38]. The composites exhibited distinct absorption bands with peak maxima at 500 nm, 496 nm, 455 nm and 410 nm for MS1, MS2, MS3 and MS4, respectively, as illustrated in Figure 3. Increasing the Ag content of samples MS1 to MS4 resulted in a shift in the absorption spectrum towards 400 nm, which corresponds to the absorption behaviour reported for pure Ag nanoparticles [38]. This shift correlates with a decrease in the magnetite phase ratio and an increase in the silver content. Additionally, the reduced overall intensity of the MS4 spectrum aligns with the XRD patterns shown in Figure 1, suggesting a significant reduction in the magnetite phase in MS4 and its substantial contribution to the absorption spectra.

Both the direct and indirect bandgap energies were calculated by plotting (hνα)^2^ and (hνα)^1/2^ as a function of the photon energy (Figure S4a,b in the Supplementary Materials) [39].

Direct and indirect band gaps for the materials reported in this study were determined and summarized in Table 3. The Fe_3_O_4_ nanoparticles have an energy bandgap of 2.5 eV, which is in good agreement with the reported values [39] for the average size determined by XRD. The formation of the composite resulted in a decrease in the redshift of the absorption wavelength and an increase in the bandgap from 2.5 eV to 3.6 and 3.7 eV. Such bandgaps allowed the catalytic activation of the composites by irradiation with the 395 nm UV LED, just below the visible spectrum, as described in Section 3.3.

### 3.3. Photodegradation Studies

#### 3.3.1. Monitoring via In Situ Luminescence Analysis

**Figure 4:**
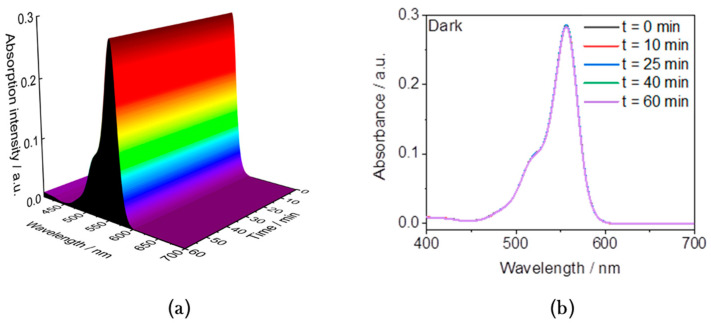
(**a**) The 3D and (**b**) 2D representations at selected reaction times of the absorption intensity of RhB (2.09 × 10^−2^ mmol/L) in the presence of H_2_O_2_ (130 mmol/L) in water and under dark conditions.

The photocatalytic activity of the prepared nanoparticles was evaluated using RhB solution (2.09 × 10^−2^ mmol/L) as a model system for organic pollutants at room temperature in the presence of H_2_O_2_ (130 mM) aqueous solution in distilled water and without pH adjustment. The nanoparticles (5 mg) were suspended and the mixture was sonicated in 15 mL of RhB solution for 15 min to reach an adsorption equilibrium. The luminescence measurements were performed after the addition of 1 mL of H_2_O_2_ solution. A first control experiment with RhB solution in the presence of H_2_O_2_ was carried out both in the dark and in the absence of a catalyst. The absorption spectrum shows a broad absorption band starting with a shoulder at 517 nm followed by a maximum at 550 nm (Figure S7). This corresponds to the typical absorption features of RhB resulting from the π → π* electronic transitions of the CN and CO groups [41]. After the addition of 1 mL of H_2_O_2_, the absorption spectra were recorded first in the dark and then at intervals of five minutes using a UV spectrophotometer to monitor the amount of dye remaining in the solution as a function of the reaction time. This reference measurement series shows only a very slight decrease in the intensity of the absorption after up to 60 min, suggesting that the degradation of the dye is slow without light irradiation and in the absence of a catalyst (Figure 4).

**Figure 5:**
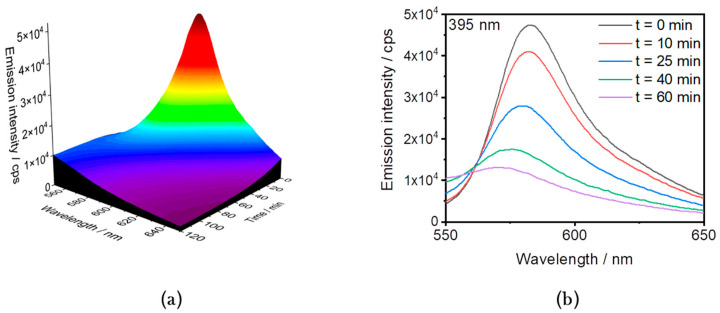
(**a**) The 3D and (**b**) 2D representations at selected reaction times of the PL of RhB (2.09 × 10^−2^ mmol/L) in the presence of H_2_O_2_ (130 mmol/L) during irradiation at 395 nm.

The same experiment was then performed under irradiation with a 395 nm UV LED. The degradation of RhB was followed by monitoring the emission of RhB with a maximum at 582 nm. Contrary to what was observed for the dark reference, the degradation of RhB was observed, as the intensity of the emission peak decreased until it disappeared completely after 2 h of irradiation (Figure 5).

As the emission intensity decreased, the maximum was shifted from 582 nm to 570 nm. The shift in the absorption peak maximum during the degradation of RhB has been reported previously and is directly related to the cleavage of ethyl groups or the oxidative degradation of the chromophore [41,42,43]. Indeed, the chemical structure of RhB consists of an aromatic core with four N-ethyl groups (Figure S6). During the degradation process of RhB by reactive oxygen species, deethylation competes with cleavage of the aromatic core [43]. When deethylation dominates, there is a significant shift in the absorption wavelength from 552 nm to 498 nm (Δ*λ* = 54 nm). This mechanism typically involves the adsorption of RhB onto the catalyst and binding via the nitrogen atoms [43]. In the absence of a catalyst, an emission shift from 582 nm to 570 nm (Δ*λ* = 12 nm) is observed during the decolourisation of RhB, consistent with the dominance of the aromatic core degradation mechanism.

Monitoring the degradation of RhB in the presence of pure Fe_3_O_4_ showed a very small increase in the degradation rate, with complete mineralisation occurring after 80 min, as opposed to 120 min for the non-catalysed system (Figure S8). The shift in the emission maximum remained constant, indicating that the cleavage of the aromatic core of RhB occurred at a faster rate than the deethylation and that the degradation rate was independent of the adsorption wavelength of the pollutant.

**Figure 6:**
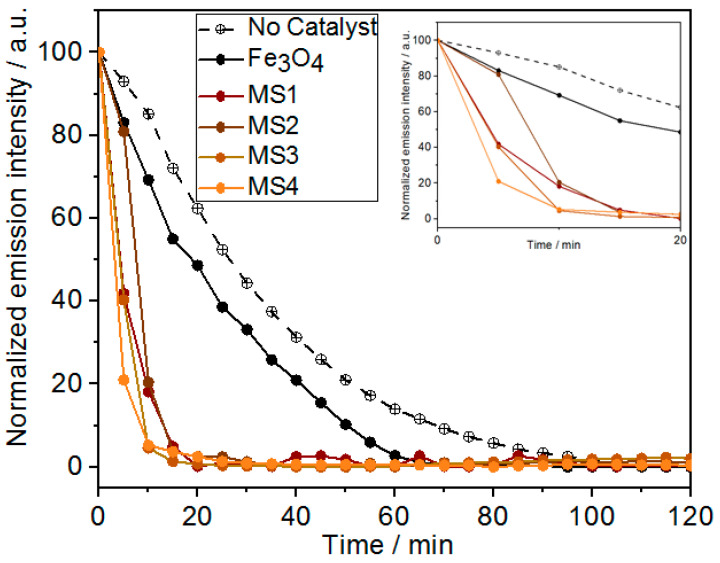
PL variation during photo-Fenton degradation of RhB (2.09 × 10^−2^ mmol/L) in the presence of H_2_O_2_ (130 mmol/L) under 395 nm irradiation without and with the prepared composite nanoparticles (MS1–4). Inset: Magnification of the first 20 min of the reaction.

In the presence of the as-prepared nanocomposites as catalysts, the degradation rate is significantly enhanced, without any significant effect of the Fe_3_O_4_/Ag ratio (Figure 6). The decolouration of RhB occurs at about t = 20 min for all four composites MS1–4 (Figure S10). The small shift from 582 nm to 570 nm is also consistent with the observations made previously on the adsorption mechanism [43].

The close examination of the luminescence changes between 0 and 20 min of the reaction (see Figure 6, inset) shows a degradation rate that increases significantly from MS1 to MS4. This trend indicates that the Ag concentration is a factor influencing the reaction, which is consistent with the possible synergy between the photocatalytic properties of Fe_3_O_4_ and a plasmonic effect within the composites.

#### 3.3.2. In Situ Monitoring of the pH Value and Redox Potential

The pH and redox potential were also monitored in situ during RhB degradation in the absence and presence of the as-prepared nanoparticles. In the absence of the catalysts, the pH of the solution increases slightly after the addition of H_2_O_2_ and during the first five minutes. However, from t = 5 min to t = 120 min, the pH value decreases continuously from 5.39 to 4.90 during the degradation of RhB. The decrease in pH can be attributed to the acidic nature of H_2_O_2_, which deprotonates to form the perhydroxyl anion ${HO}_{2}^{-}$ (see Equation (2)) [44]. The slow decrease in luminescence suggests a slow rate of photodecomposition of H_2_O_2_ or its conjugated base, the hydroperoxide anion $HO_{2}^{-}$, into ROS, such as the hydroxyl radical (•OH) or the perhydroxyl radical (${•O}_{2}H$) (see Equation (3)). These reactions usually require activation at very short wavelengths (from 240 nm to 280 nm) [45] and cannot be achieved with UV LEDs [46].

(2) $$H_{2}O_{2}\to HO_{2}^{-}+H^{+}$$

(3) $$HO_{2}^{-}+H_{2}O_{2}\overset{h\nu \;}{\to }•OH+{•O}_{2}H+{OH}^{-}$$

(4) $${Fe}^{2+}+H_{2}O_{2}\overset{h\nu }{\to }{{Fe}^{3+}+}^{\;}HO•$$

In the presence of Fe_3_O_4_ nanoparticles as catalysts, the pH decrease is more pronounced, from 6.81 to 4.77, in agreement with the participation of Fe_3_O_4_ in its decomposition, most probably through the Haber–Weiss mechanism (Equation (4)) [47]. The slight decrease after t = 60 min and after the decomposition, detected by the in situ monitoring of the PL, could explain the final mineralisation of the organic fragment and the in situ formation of carbonic acid (Equations (5) and (6)).

(5) $$•OH+\mathrm{RhB}\;\to CO_{2}+H_{2}O$$

(6) $${CO}_{2}+H_{2}O\to HCO_{3}^{-}+H^{+}$$

The redox potential increases slowly from 324 mV to 374 mV in the absence of a catalyst. This slow increase is consistent with the decomposition of H_2_O_2_ and the photocatalytic production of the hydroxyl radical. In contrast, in the presence of Fe_3_O_4_, the redox potential decreases until t = 20 min, indicating the slightly faster consumption of ROS and the obvious photocatalytic activity of Fe_3_O_4_ nanoparticles. In fact, Fe^2+^ cations can catalyse the formation of the hydroxyl radical (Fenton reaction). Therefore, the faster degradation of RhB in the presence of Fe_3_O_4_ than without the catalyst was expected. However, Figure 6 shows a moderate difference in the rate of degradation compared to the same process in the absence of a catalyst. This is consistent with the moderate catalytic activity of the iron species at a pH above 3 [8].

For the reactions with Fe_3_O_4_/Ag nanocomposites, the acceleration of the dye degradation, as already demonstrated by the in situ luminescence measurements, is reflected in a very strong increase in the pH only a few minutes after the addition of the H_2_O_2_ solution. Such a significant and rapid change can occur during the decomposition of H_2_O_2_ initially adsorbed on the surfaces of the active sites of the catalyst [47]. These variations are more than ten times higher than those observed in the presence of the Fe_3_O_4_ nanoparticle catalyst. Such pH increases have already been reported [48] as a possible consequence of the production of ${OH}^{-}$ ions and of the local increase in temperature. It is important to note that the lower initial pH of the solutions compared to that observed for Fe_3_O_4_ could be one of the reasons for the high decomposition rate of RhB. In fact, the Fenton process occurs preferentially at low pH values [8,49].

The greatest and fastest increase in pH is obtained with MS2 as a catalyst during the photo-Fenton reaction and corresponds to the fastest drop in the redox potential values, from 337 mV to 215 mV after only 15 min. This decrease is due to the accelerated production and consumption of hydroxyl radicals. The latter are powerful oxidants whose presence leads to an overall increase in the oxidative capacity of a system [50]. After this sharp decrease in the redox potential values, more than 95% of the dye emission is eliminated when RhB is oxidised in the presence of Fe_3_O_4_/Ag heterostructures. After this initial phase, the redox potential increases in a similar manner to that observed both in the absence of catalysts and in the presence of Fe_3_O_4_ nanoparticles. This behaviour is consistent with the increase in pH, which is responsible for the mineralisation of RhB and the generation of carbonic acid [51]. Finally, the plateau observed indicates the end of the mineralisation response.

The trend towards an overall increase in the degradation rate of RhB in the presence of MSx nanocomposites could further be attributed to the morphological and electronic effects of the heterostructures. On the one hand, the formation of biphasic composites prevents the strong aggregation of tiny Fe_3_O_4_ nanoparticles by dispersing them on the surfaces of larger silver nanoparticles. On the other hand, the silver phase, whose diameter is less than 50 nm, exhibits a plasmonic absorption band close to 395 nm. After irradiation, there is a significant increase in the production rate and concentration of the hydroxyl radical in relation to the variation in the redox potential. The latter is highly reactive and the degradation of RhB is therefore very rapid.

A mechanism for the degradation of RhB, considering the results obtained here, is proposed below. In the absence of the Fe_3_O_4_/Ag catalyst or in the presence of Fe_3_O_4_ nanoparticles, the direct splitting of H_2_O_2_ or its conjugated base ${HO}_{2}^{-}$ is initiated solely by the absorption of photons.

According to the literature [50], the degradation of the dye results from the reaction of the •OH radical with the aromatic core until its decomposition and mineralisation. The formation of other reactive oxygen species, such as the superoxide radical (•$O_{2}^{-}$ or •O_2_H), occurs via the oxidation of the hydroxyl radical (Equations (4) and (5)), but these other species are less reactive than the hydroxyl radical.

**Figure 7:**
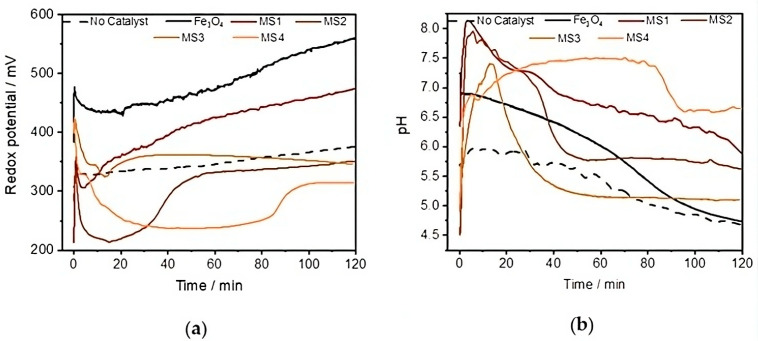
In situ variation in (**a**) pH and (**b**) redox potential of 15 mL RhB solution (2.09 × 10^−2^ mmol/L) by 1 mL H_2_O_2_ (130 mmol/L) in the absence and presence of 5 mg Fe_3_O_4_ nanoparticles and nanocomposites.

In the presence of the nanocomposite particles, the LSPR enhances the splitting of H_2_O_2_ to such an extent that it undergoes very little deprotonation, as shown by the pH variation in Figure 7b. Instead, the acidic compound is rapidly converted into the hydroxyl radical and hydroxyl anion following the absorption of light by the catalyst and the formation of a reactive electron–hole pair. The hydroxyl radical has higher oxidation potential and therefore higher reactivity towards substrates [50].

(7) $$H_{2}O_{2}+e^{-}\overset{h\nu }{\to }•OH+HO^{-}$$

The ROS formed in the solution react rapidly with the organic dye in successive degradation reactions, including intermediates, to form CO_2_ and H_2_O as end products.

#### 3.3.3. Recovery and Reuse of Catalytic Nanocomposite

The aim of this work on the formation of Fe_3_O_4_/Ag nanocomposites for photo-Fenton catalysis was not only to tune the absorption region and increase the reaction, but also to achieve easy catalyst regeneration as part of the intended objectives. In heterogeneous catalysis, the separation of the catalyst from the treated effluent is usually time-consuming and operationally complex, e.g., via filtration [52]. Due to the magnetic behaviour of the particles, it is possible to separate them from the liquid medium using a magnet within a few minutes. Magnetically mediated separation was performed on the Fe_3_O_4_/Ag nanocomposites after one cycle of RhB degradation. The separation efficiency decreased significantly as the silver content of the particles increased. MS1 nanocomposites could be completely separated after 2 min of exposure to the magnet. The separation of MS2 from the effluent took 5 min. MS3 and MS4 could not be completely removed after 30 min of treatment, as the solution still exhibited the pale pink and bluish colors characteristic of plasmonic nanoparticles (Figure S10).

**Figure 8:**
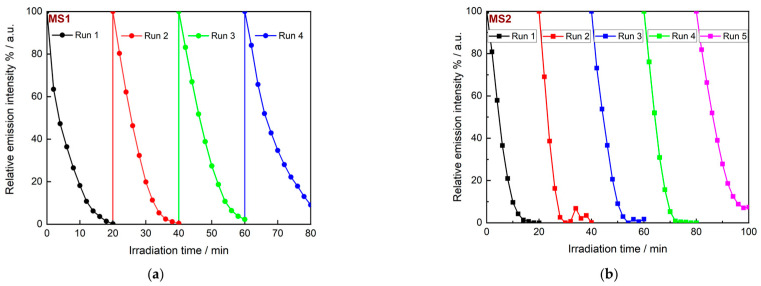
Cyclic experiments on MS1 (**a**) and MS2 (**b**) nanocomposites. Photocatalytic conditions: 50 mg of catalyst with 150 mL of RhB solution (2.09 × 10^−2^ mmol/L).

Several successive catalytic cycles were also carried out with MS1 and MS2, as they were the most promising in terms of easy and rapid recovery. At the end of each cycle and after the complete mineralisation of RhB, the particles were separated after 20 min of contact of the flask with a magnet, washed twice with water and then added to fresh solutions of the dye. The PL was monitored again and the various experiments showed that the rate of degradation slowed down for both samples. However, the photocatalytic degradation was still rapid, with a quasi-quantitative yield of 90% for MS1 after 20 min (Figure 8a). When MS2 was used as catalyst, the complete decolourisation of the dye was even faster after about 15 min. This trend was maintained for at least five cycles.

## 4. Conclusions

A series of Fe_3_O_4_/Ag nanocomposite materials with increasing Ag content has been prepared using the co-precipitation method. The composites consist of nano-sized heterostructures in which Ag nanoparticles are decorated with smaller Fe_3_O_4_ nanoparticles. The composites’ magnetic properties enable the separation of the particles from the mixture in the solution via the application of an external magnetic field. The formation of composite nanoparticles with equimolar content of Fe and Ag results in a bi-phasic material with a large specific surface area and therefore important potential for the adsorption of pollutants. RhB degradation was performed in a photo-Fenton-like oxidation using magnetite and composite particles. In situ monitoring of the emission intensity shows that the reactivity of H_2_O_2_ is scarcely affected by the presence of Fe_3_O_4_, while all Ag-containing composites are able to reduce the RhB degradation time from one hour to 15 min. In situ monitoring of the pH and redox potential shows that the catalysis is due to the rapid production of •OH as ROS at the beginning of the reaction. The overall observations are consistent with a photo-Fenton mechanism in which the oxidative degradation of the bulk chromophore dominates, and they provide indications regarding the nature of the chemical transformations during conversion. Furthermore, the approach of using a simple setup and accessible laboratory techniques for in situ monitoring could be applied to other photocatalytic systems or combined with complementary in situ techniques. In particular, to elucidate further the surface chemistry and catalytic mechanisms involved, the X-ray photoelectron spectroscopy (XPS) analysis of the catalysts after the reaction is proposed. This will enable a more detailed investigation into the oxidation states and surface functionalities that contribute to the enhanced catalytic activity observed in Ag-containing composites.

## Figures and Tables

**Table 1:** Summary of the crystallite size, lattice strain and relative quantitative molar ratio of the Fe_3_O_4_ and Ag phases.

Compound	Crystallite Size (nm)		Lattice Strain	Calculated Phase Ratio (%)	
	Average Value	Standard Dev.	Plane (111)	Fe_3_O_4_	Ag
Fe_3_O_4_	19	2	0.0039	100	0
MS1	24	5	0.0082	52	48
MS2	30	7	0.0056	34	66
MS3	38	14	0.0046	21	79
MS4	39	7	0.0042	11	89

**Table 2:** Values of the hydrodynamic size (D_H_), polydispersity index (PDI) and zeta potential (ζ) determined by DLS measurements.

Compound	D_H_ (nm)	PDI	ζ-Potential (mV)
Fe_3_O_4_	406	0.182	2.9
MS1	98	0.265	−6.2
MS2	118.3	0.246	−7.6
MS3	121.7	0.278	−2.2
MS4	213.3	0.294	−9.0

**Table 3:** Summary of the direct and indirect bandgaps of the synthesised nanoparticles.

Sample	Direct Bandgap (eV)	Indirect Bandgap (eV)
Fe_3_O_4_	2.5	1.8
MS1	3.7	3.7
MS2	3.7	3.7
MS3	3.6	3.7
MS4	3.6	3.7

## Data Availability

All data presented in this work are available on reasonable request from the corresponding authors.

## Supplementary Materials

The following supporting information can be downloaded at: https://www.mdpi.com/article/10.3390/nano14100849/s1, Figure S1: SEM images of (a) M1, (b) MS2, (c) MS3 and (d) MS4 nanocomposites; Figure S2: Size distribution of aqueous solution containing (a) Fe_3_O_4_, (b) MS1, (c) MS2, (d) MS3 and (e) MS4 sonicated for 15 min without pH adjustment or surfactant addition; Figure S3: (a) Infrared and (b) Raman superimposed spectra of Fe_3_O_4_ and MSx composites; Figure S4a: Tauc’s plot and extrapolation for the determination of the direct and indirect bandgaps of the particles; Figure S4b: Tauc’s plot and extrapolation for the determination of the direct and indirect bandgaps of the particles; Figure S5a: X-ray diffraction patterns of Fe_3_O_4_ illustrating the five most prominent reflexes selected for the calculation of the crystallite size; Figure S5b: X-ray diffraction patterns of MS1 illustrating the five most prominent reflexes selected for the calculation of the crystallite size; Figure S5c: X-ray diffraction patterns of MS2 illustrating the five most prominent reflexes selected for the calculation of the crystallite size; Figure S5d: X-ray diffraction patterns of MS3 illustrating the five most prominent reflexes selected for the calculation of the crystallite size; Figure S5e: X-ray diffraction patterns of MS4 illustrating the five most prominent reflexes selected for the calculation of the crystallite size; Figure S6: Chemical structure of RhB showing the four N-ethyl groups; Figure S7: Variation in the absorbance of RhB (2.09 × 10^−2^ mmol/L) with H_2_O_2_ (130 mmol/L) in water in dark conditions (a) without a catalyst and (b) in the presence of Fe_3_O_4_ nanoparticles; Figure S8: Variation in the absorption intensity of RhB (2.09 × 10^−2^ mmol/L) in the presence of H_2_O_2_ under (a) dark conditions and (b) under irradiation at 395 nm for selected reaction times; Figure S9: Photographs of the reactor before (a), during (b) and after (c) the degradation of RhB by H_2_O_2_ and MS2 under UV (395 nm) irradiation; Figure S10: Photographs of samples after RhB degradation and upon magnet application; Figure S11: Nitrogen adsorption-desorption isotherms for MS1 (a), MS2 (b), MS3 (c) and MS4 (d); Figure S12: EDX spectra of MS1, MS2, MS3 and MS4 nanocoposites; Table S1: The average crystallite size of Fe_3_O_4_ calculated by Scherrer’s equation; Table S2: The average crystallite size of MS1 calculated by Scherrer’s equation; Table S3: The average crystallite size of MS2 calculated by Scherrer’s equation; Table S4: The average crystallite size of MS3 calculated by Scherrer’s equation; Table S5: The average crystallite size of MS4 calculated by Scherrer’s equation; Table S6: Summary of the data obtained from BET measurements; Table S7: Atomic percentages obtained from the EDX analysis of the 4 composites.
