# Supplementary material for: Towards High-Performance Photo-Fenton Degradation of Organic Pollutants with Magnetite-Silver Composites: Synthesis, Catalytic Reactions and In Situ Insights
Source: Nanomaterials (Basel). 2024 May 13;14(10):849. doi: 10.3390/nano14100849 (PMC11124028; doi:10.3390/nano14100849)
Supplement: Supplementary file 1 [file nanomaterials-14-00849-s001.zip › nanomaterials-2974908-supplementary.pdf]

Towards High-Performance Photo-Fenton Degradation of Organic Pollutants with Magnetite-Silver Composites: Synthesis, Catalytic Reactions and In Situ Insights

Katia Nchimi Nono [1,2,*], Alexander Vahl [3,4] and Huayna Terraschke [1,4,*]

1 Institute of Inorganic Chemistry, Kiel University, Max-Eyth-Str. 2, 24118 Kiel, Germany
2 Department of Inorganic Chemistry, Faculty of Science, University of Yaoundé, Yaoundé P.O. Box 812, Cameroon
3 Department for Material Science, Kiel University, Kaiserstr. 2, 24143 Kiel, Germany
4 Kiel Nano, Surface and Interface Science (KiNSIS), Christian-Albrechts-Platz 4, 24118 Kiel, Germany
* Correspondence: knchiminono@ac.uni-kiel.de (K.N.N.); hterraschke@ac.uni-kiel.de (H.T.); Tel.: +49-0431-880-2988 (K.N.N.)

Electronic supporting information

Contents

1. SEM images of MS1, MS2, MS3 and MS4 nanoparticles 2
2. DLS measurements of the hydrodynamic size of Fe_3O_4, MS1, MS2, MS3 and MS4 nanoparticles 3
3. FTIR and Raman spectra of the nanocomposites 4
4. Tauc's plot obtained for the synthesized compounds 5
5. Crystallite size calculation 6
6. Structural representation of RhB 9
7. Variation of the absorbance of RhB in the presence H_2O_2 in dark conditions 10
8. 2D and 3D variation of the photoluminescence during photocatalytic degradation of RhB 11
9. Photographs of the setup of the monitoring of the pH value, redox potential and photoluminescence during the degradation of RhB in the presence of H_2O_2 under UV (395 nm) irradiation 12
10. Specific surface area determination 13
11. N_2 BET adsorption analysis 14
12. EDX elemental analysis 15

1. SEM images of MS1, MS2, MS3 and MS4 nanoparticles

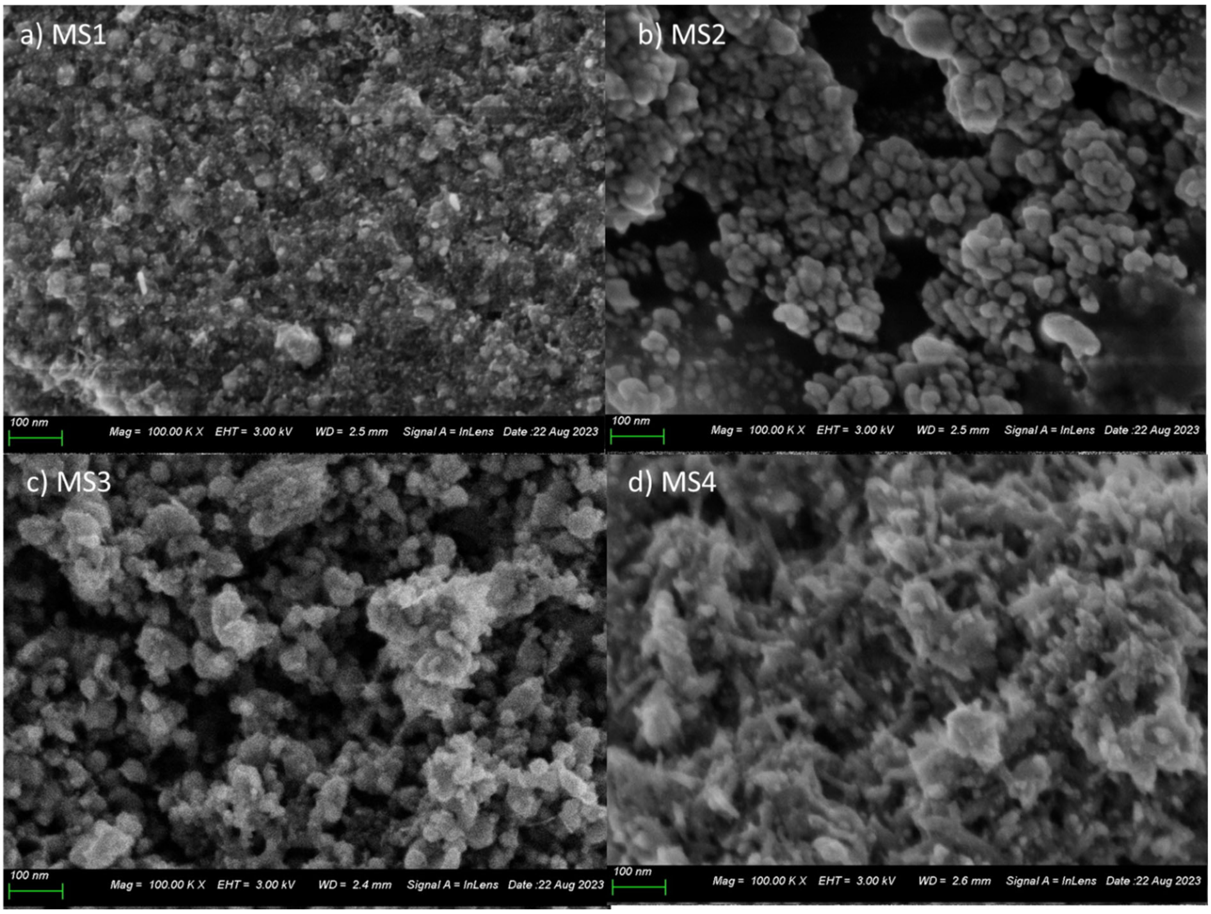

Figure S1: SEM images of (a) M1, (b) MS2, (c) MS3 and (d) MS4 nanocomposites.

2. DLS measurements of the hydrodynamic size of Fe_3O_4, MS1, MS2, MS3 and MS4 nanoparticles

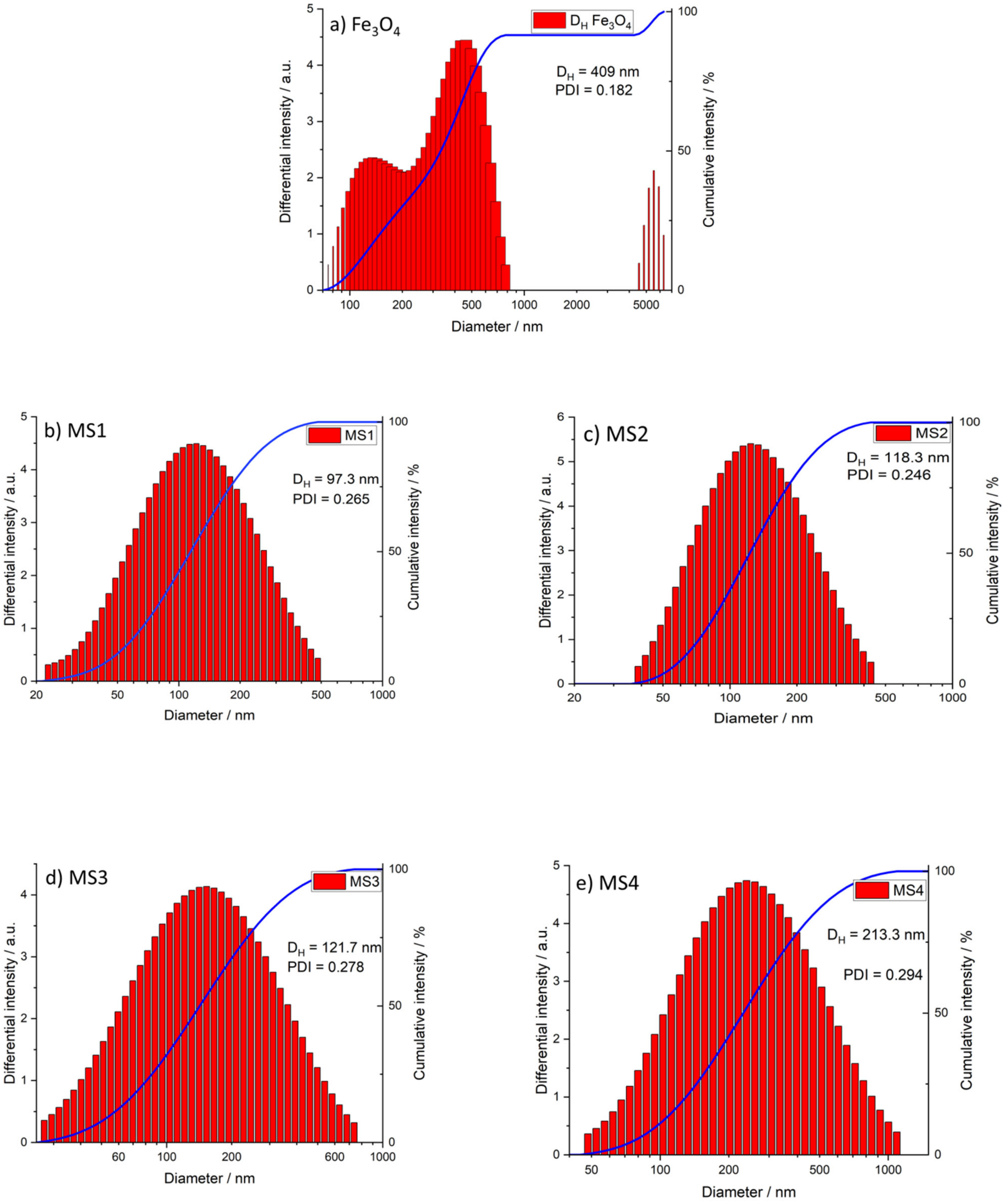

Figure S2: Size distribution of aqueous solution containing (a) Fe_3O_4, (b) MS1, (c) MS2, (d) MS3 and (e) MS4 sonicated for 15 min without pH adjustment or surfactant addition.

3. FTIR and Raman spectra of the nanocomposites

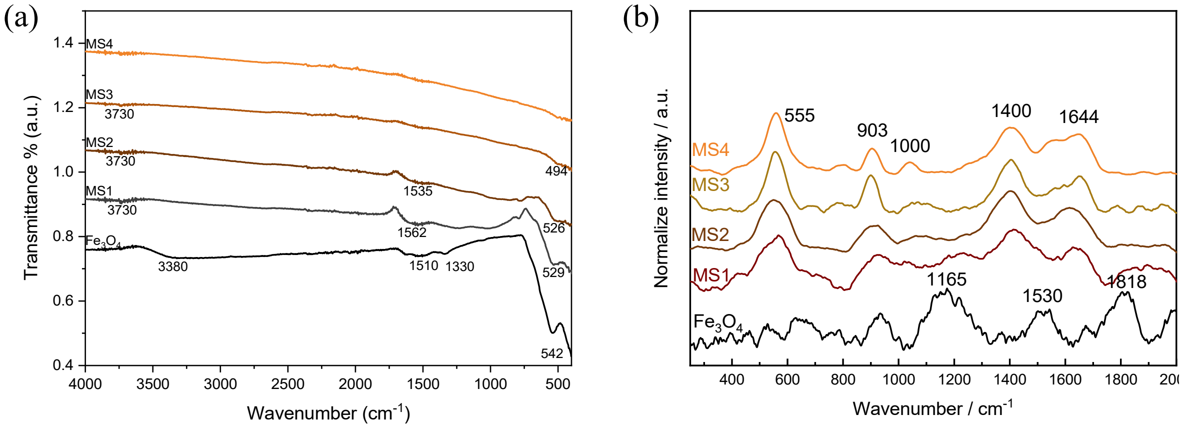

Figure S3: (a) Infrared and (b) Raman superimposed spectra of Fe_3O_4 and MSx composites.

4. Tauc's plot obtained for the synthesized compounds

a) Direct bandgap

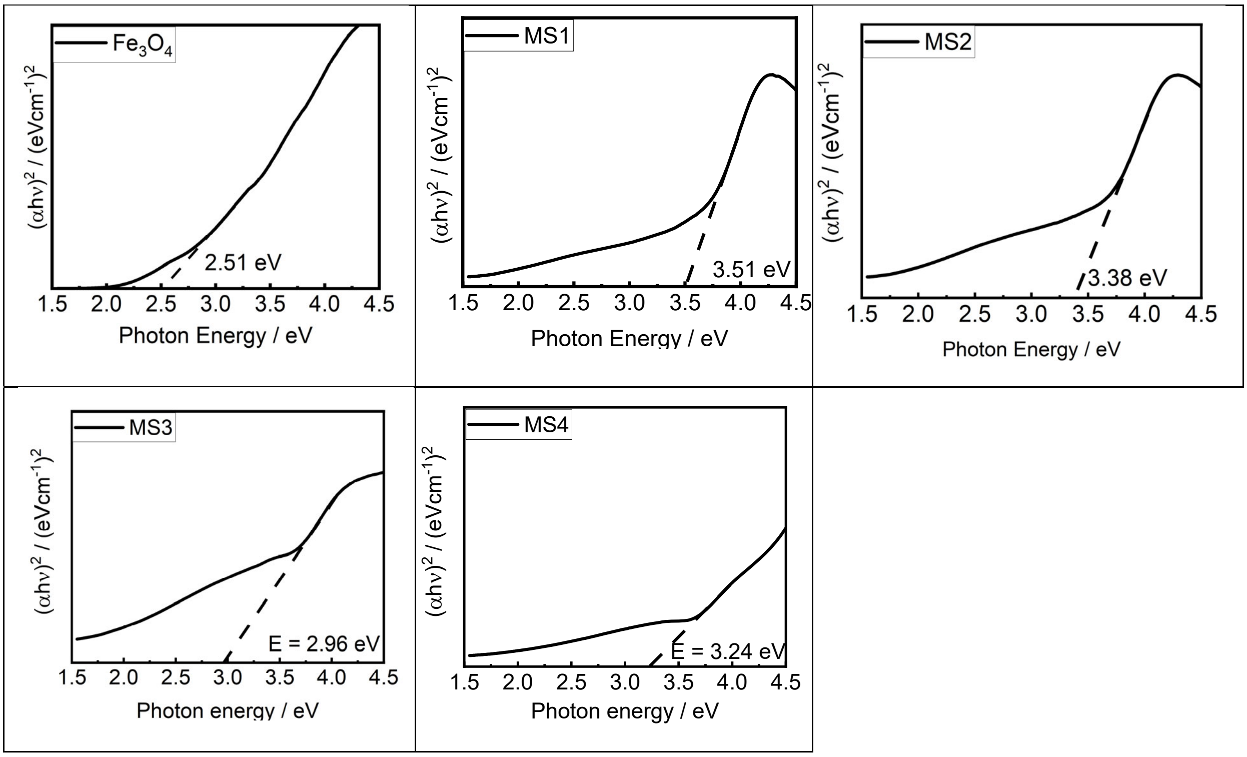

b) Indirect bandgap

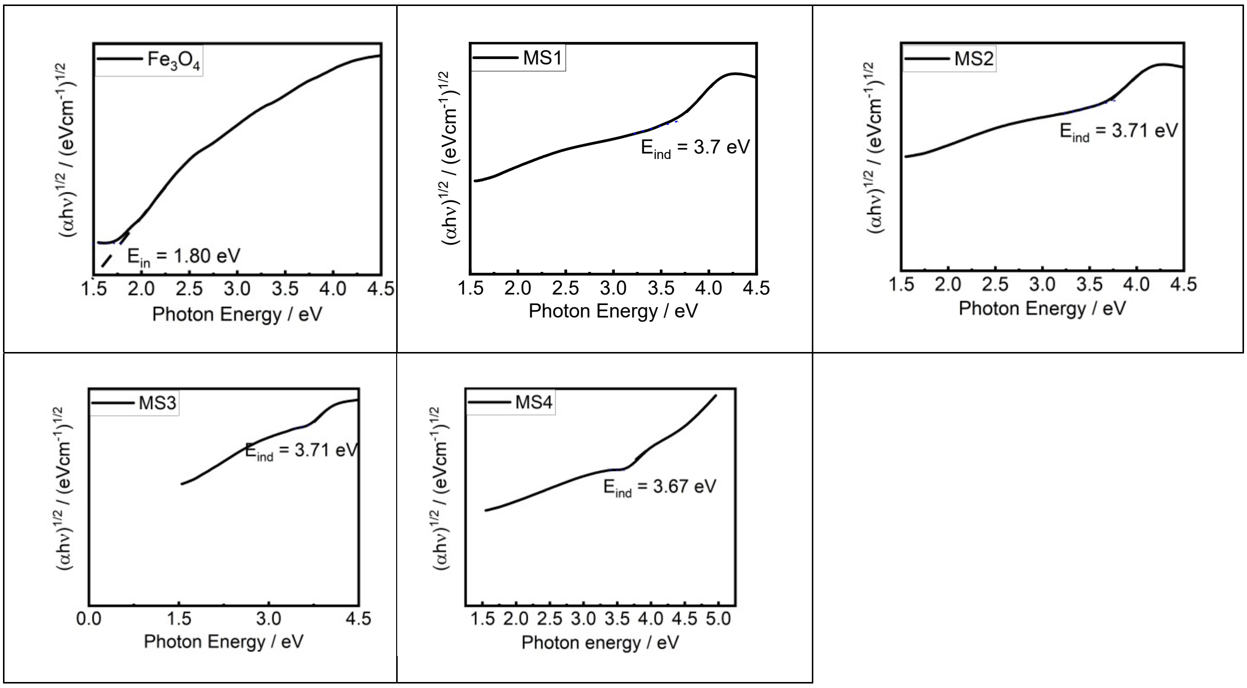

Figure S4: Tauc's plot and extrapolation for the determination of the a) direct and b) indirect bandgaps of the particles.

5. Crystallite size calculation

Scherrer's equation is used for determining the crystallite size of nanoparticles from X-ray diffraction (XRD) data. The equation relates the peak width of a diffraction peak to the size of the crystallites that caused the peak. The equation is given as:

$D = K\lambda/\beta \cos \theta$

where D is the crystallite size.
K: Scherrer constant (taken to be equal to 0.9)
λ: X-ray wavelength (for Mo, λ = 0.70930),
β: full width at half maximum (FWHM) of the diffraction peak
θ: Bragg angle.

For each compound, FWHM is measured after baseline correction and linear fitting of the peaks on ORIGIN software. The crystallite size for the most intense peaks of each phase were calculated and presented in tables below.

Table S1: The average crystallite size of Fe_3O_4 calculated by Scherrer´s equation.

2θ	θ (°)	θ (rad)	cos(θ)	β=FWHM	β (rad)	D / nm	Dm / nm	Stdr. dev.
13.737	6.868	0.120	0.993	0.389	0.007	20	19	2
16.135	8.068	0.141	0.990	0.419	0.007	19		
19.524	9.762	0.170	0.986	0.457	0.008	17		
25.437	12.718	0.222	0.975	0.394	0.007	20		
27.746	13.873	0.242	0.971	0.407	0.007	20		

Table S2 : The average crystallite size of MS1 calculated by Scherrer´s equation.

2θ	θ (°)	θ (rad)	cos(θ)	β=FWHM	β (rad)	d / nm	d_m / nm	Dev.
16.098	8.049	0.140	0.990	0.489	0.009	16	24	5
17.248	8.624	0.150	0.989	0.269	0.005	30		
19.941	9.970	0.174	0.985	0.351	0.006	23		
28.392	14.196	0.248	0.969	0.299	0.005	27		
33.423	16.711	0.292	0.958	0.312	0.005	26		

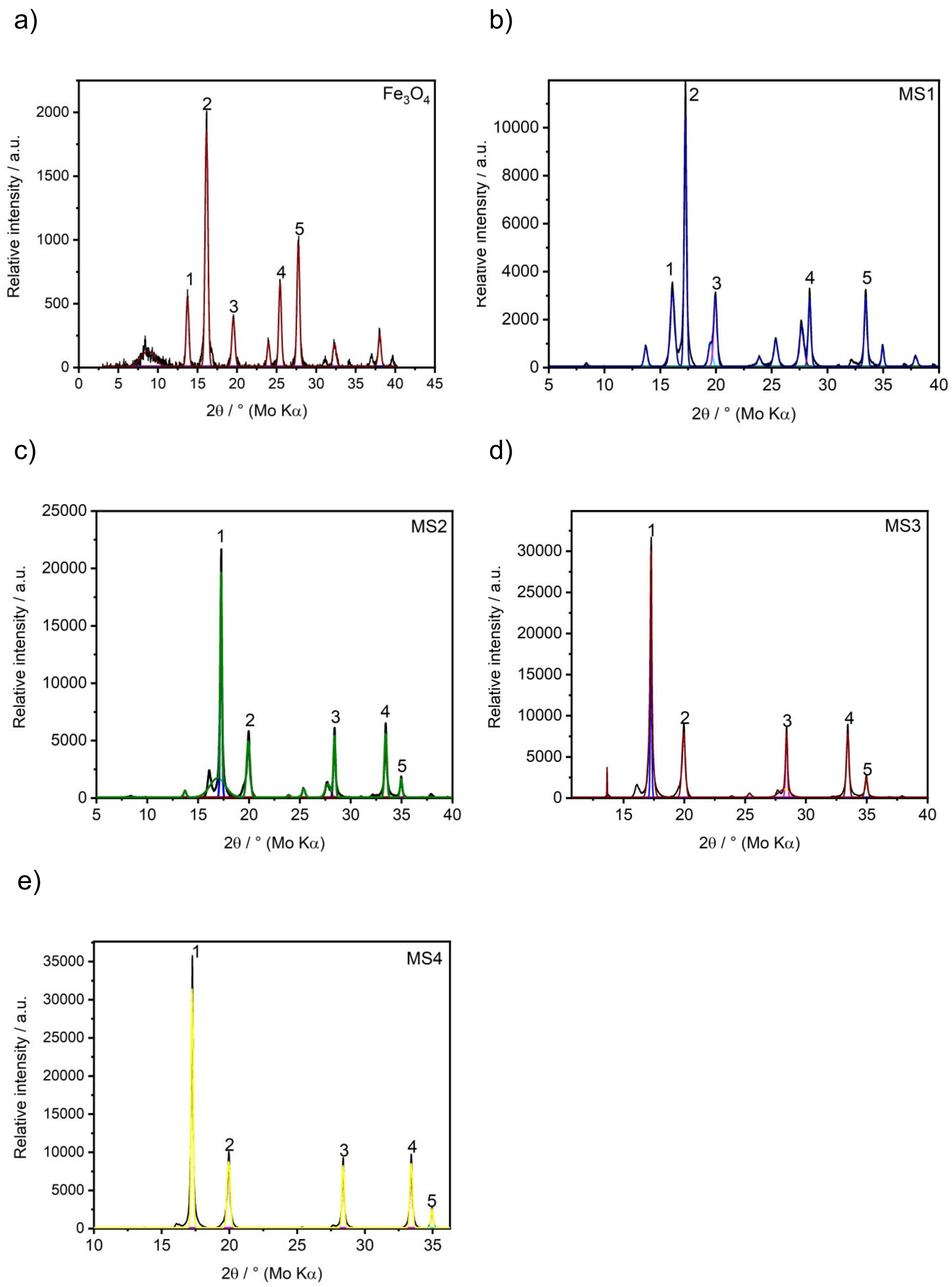

Figure S5 : X-ray diffraction patterns of a) Fe_3O_4 b) MS1, c) MS2, d) MS3, e) MS4 illustrating the five most prominent reflexes selected for the calculation of the crystallite size.

Table S3 : The average crystallite size of MS2 calculated by Scherrer´s equation.

2θ	θ (°)	θ (rad)	cos(θ)	β=FWHM	β (rad)	d / nm	d_m / nm	Dev.
17.254	8.627	0.150	0.989	0.206	0.004	39	30	7
19.927	9.964	0.174	0.985	0.404	0.007	20		
28.400	14.200	0.248	0.969	0.243	0.004	33		
33.428	16.714	0.292	0.958	0.284	0.005	29		
34.953	17.476	0.305	0.954	0.268	0.005	31		

Table S4 : The average crystallite size of MS3 calculated by Scherrer´s equation.

2θ	θ (°)	θ (rad)	cos(θ)	β=FWHM	β (rad)	d / nm	dm / nm	Dev.
17.259	8.629	0.151	0.989	0.134	0.002	59	38	14
19.938	9.969	0.174	0.985	0.330	0.006	24		
28.397	14.198	0.248	0.969	0.188	0.003	43		
33.428	16.714	0.292	0.958	0.269	0.005	31		
34.955	17.478	0.305	0.954	0.260	0.005	32		

Table S5 : The average crystallite size of MS4 calculated by Scherrer´s equation.

2θ	θ (°)	θ (rad)	cos(θ)	β=FWHM	β (rad)	d / nm	d_m / nm	Dev.
17.253	8.626	0.150	0.989	0.182	0.003	44	39	7
19.943	9.971	0.174	0.985	0.275	0.005	29		
28.389	14.195	0.248	0.969	0.198	0.003	41		
33.418	16.709	0.291	0.958	0.218	0.004	38		
34.950	17.475	0.305	0.954	0.179	0.003	46		

6. Structural representation of RhB

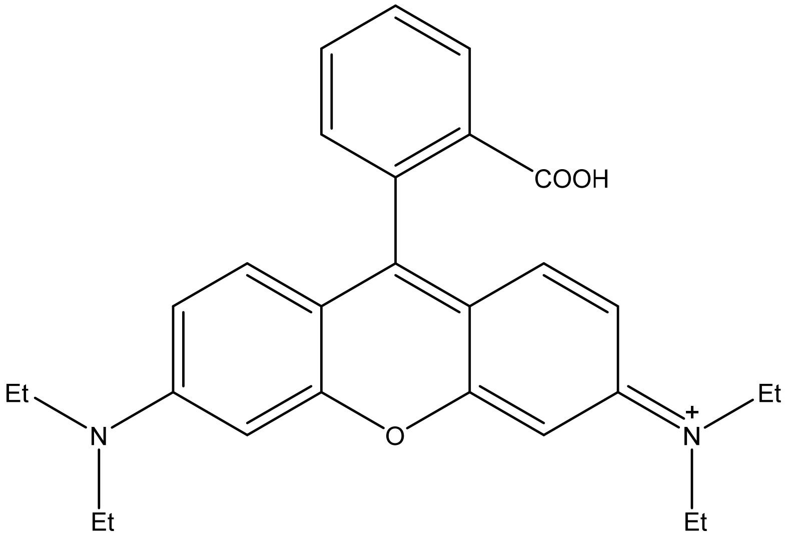

Figure S6 : Chemical structure of RhB showing the four N-ethyl groups.

7. Variation of the absorbance of RhB in the presence H_2O_2 in dark conditions

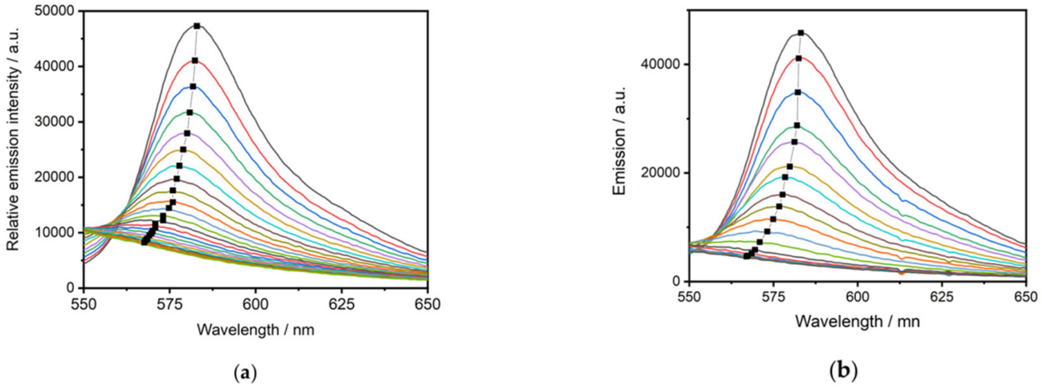

Figure S7: Variation in the absorbance of RhB (2.09 × 10^{-2} mmol/L) with H_2O_2 (130 mmol/L) in water in dark conditions (a) without a catalyst and (b) in the presence of Fe_3O_4 nanoparticles.

8. 2D and 3D variation of the photoluminescence during photocatalytic degradation of RhB

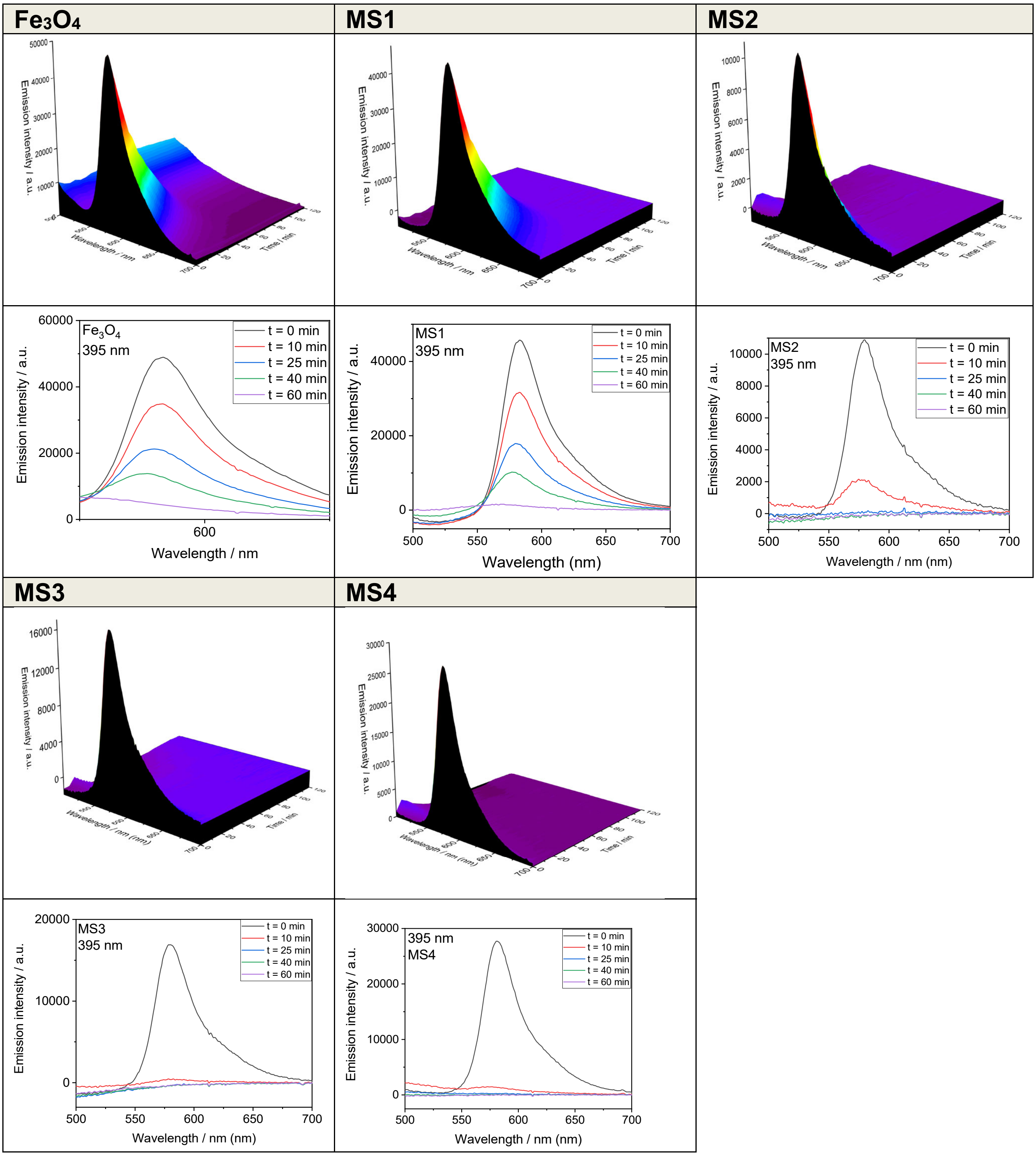

Figure S8 : Variation in the absorption intensity of RhB (2.09×10^{-2} mmol/L) in the presence of H_2O_2 under (a) dark conditions and (b) under irradiation at 395 nm for selected reaction times.

9. Photographs of the setup of the monitoring of the pH value, redox potential and photoluminescence during the degradation of RhB in the presence of H_2O_2 under UV (395 nm) irradiation.

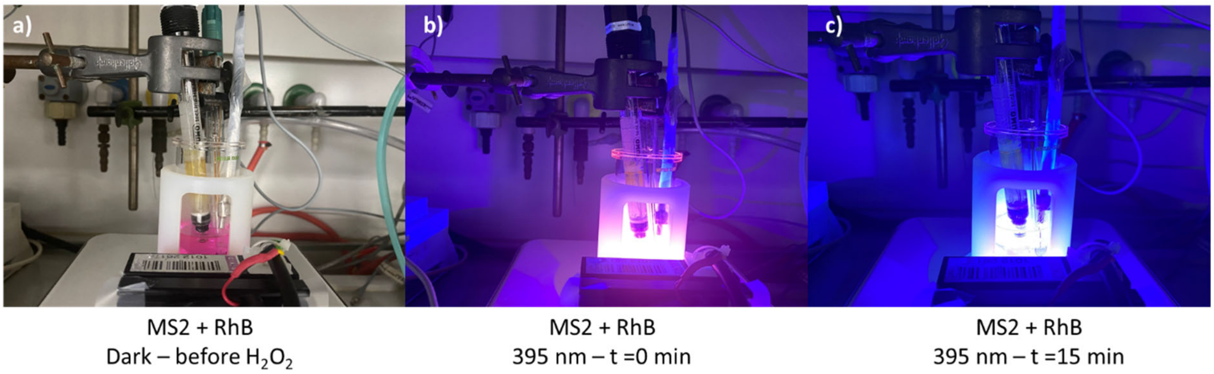

Figure S9 : Photographs of the reactor before (a), during (b) and after (c) the degradation of RhB by H_2O_2 and MS2 under UV (395 nm) irradiation.

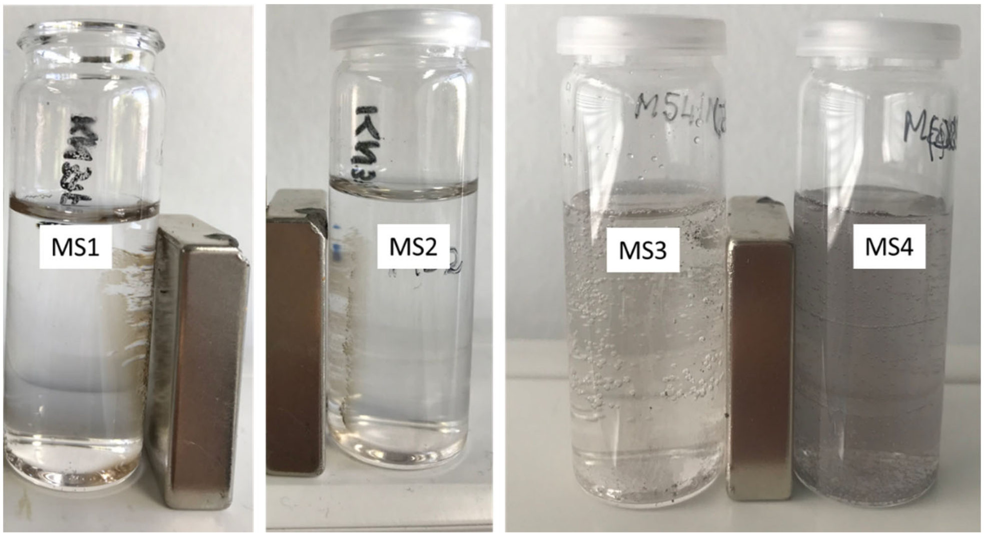

Figure S10 : Photographs of samples after RhB degradation and upon magnet application.

10. Specific surface area determination

Table S6 : Summary of the data obtained from BET measurements.

**Compound**	**Fe_3O_4**	**MS1**	**MS2**	**MS3**	**MS4**
Specific surface area ($m^2 . g^{-1}$)	nd	126.31	79.66	39.33	18.95
Average diameter of pores (nm)	nd	2.6	5.3	8.9	14.1

11. N_2 BET adsorption analysis

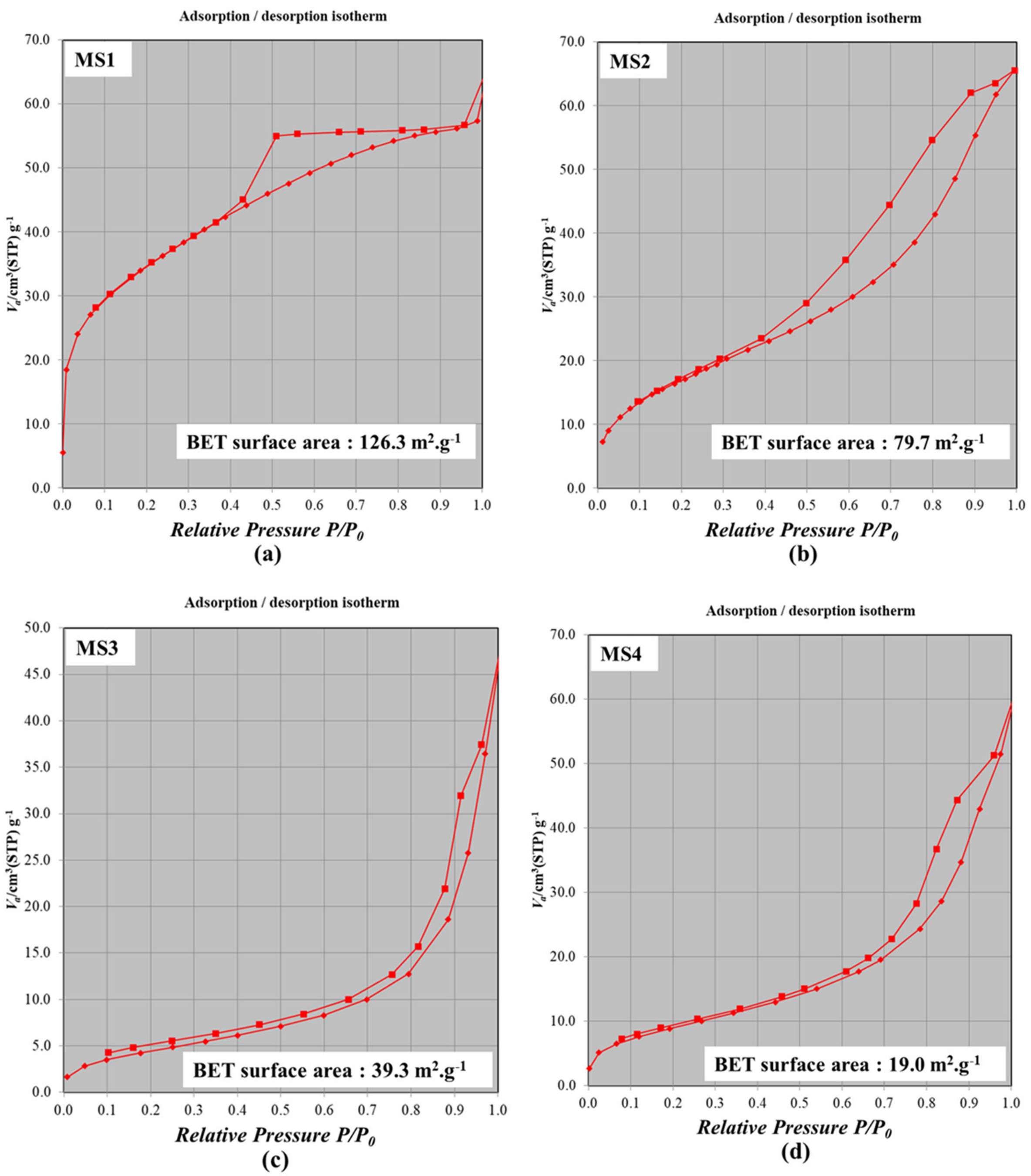

Figure S11: Nitrogen adsorption-desorption isotherms for MS1 (a), MS2 (b), MS3 (c) and MS4 (d).

12. EDX elemental analysis

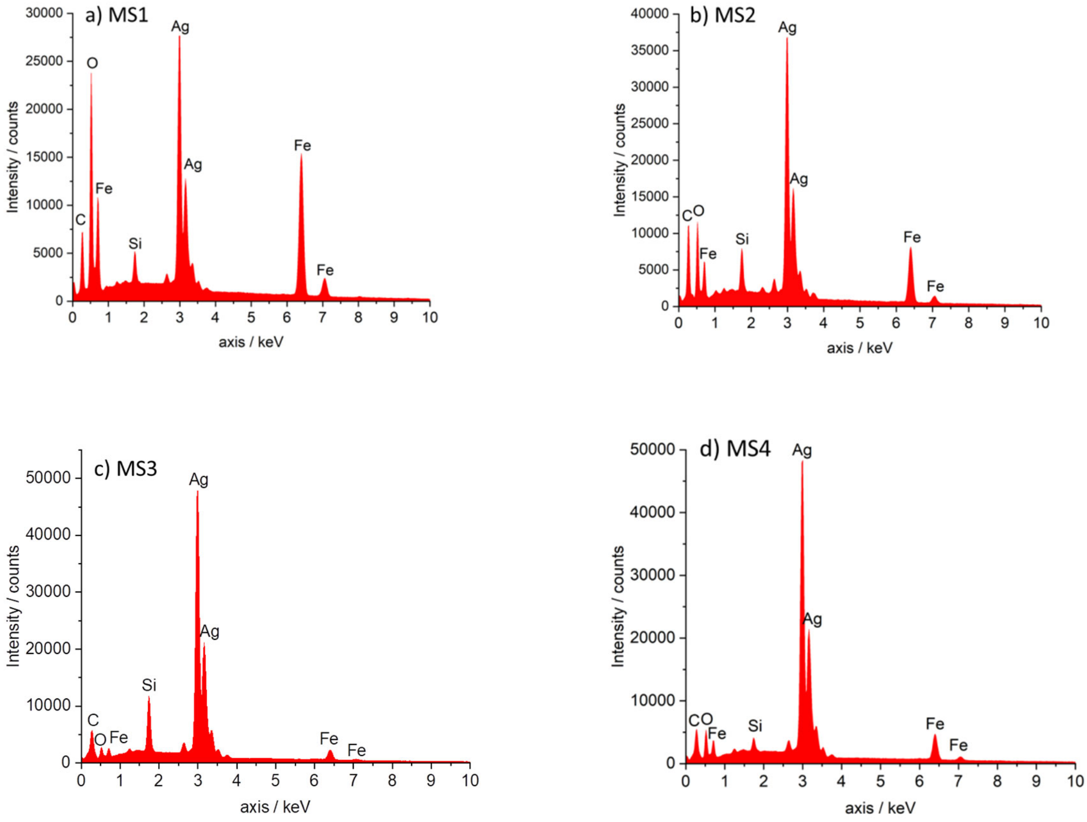

Figure S12 :EDX spectra of MS1, MS2, MS3 and MS4 nanocoposites.

Table S7: Atomic percentages obtained from the EDX analysis of the 4 composites.

	Atomic percent							
**Compound**	**C**	**O**	**Si**	**Fe**	**Br**	**Ag**	**Total**	**Ag/Fe Ratio**
**MS1**	17.88	43.58	1.46	24.92		12.15	100	0.5
**MS2**	28.79	33.4	3.03	15.33		19.46	100	1.2
**MS3**	15.85	17.07	9.97	6.77		50.33	100	7.5
**MS4**	13.59	30.01	1.79	13.11	0.18	41.32	100	3.1
